# The SNARE protein Ykt6 drives insertion of the GluA1 and GluA2 glutamate receptors at synaptic spines during long-term potentiation

**DOI:** 10.1016/j.jbc.2025.110613

**Published:** 2025-08-19

**Authors:** Momoko Takahashi, Maya Raman, Gabriela Caraveo

**Affiliations:** Department of Neurology, Feinberg School of Medicine, Northwestern University, Chicago, Illinois, USA

**Keywords:** glutamate receptor, long-term potentiation, hippocampus, SNARE, Ykt6, ER, Golgi

## Abstract

Long-term potentiation (LTP), a crucial form of synaptic plasticity essential for memory and learning, depends on protein synthesis and the upregulation of GluA1 and GluA2 at postsynaptic compartments. While extensive research has focused on the role of endosomal trafficking in GluA1 and GluA2 regulation, the contribution of the secretory pathway, namely endoplasmic reticulum, Golgi trafficking pathways remains largely unexplored. A key opportunity to investigate this emerged from Ykt6, an evolutionarily conserved SNARE protein and a master regulator of vesicular fusion along the secretory pathway. Here, we demonstrate that Ykt6 is highly expressed in the mammalian hippocampus, localizes to synaptic spines where it regulates GluA1 and GluA2 surface expression in an LTP-dependent manner. Furthermore, we found that Ykt6 modulates spine morphology, synaptic vesicle pool dynamics, as well as the amplitude and frequency of miniature excitatory postsynaptic currents. Ykt6 loss of function has been linked to α-synuclein pathology, a hallmark of Lewy body dementias, where α-synuclein misfolding in the hippocampus disrupts LTP. Collectively, our findings identify Ykt6 as a key SNARE protein supporting hippocampal function and LTP, with potential relevance to the pathogenesis of LBDs.

Through an unbiased phosphoproteomic approach, our laboratory previously identified Ykt6 as a substrate of the Ca^2+^-dependent serine/threonine phosphatase, calcineurin (CaN) (1). Ykt6, an essential SNARE, regulates vesicular fusion along the secretory pathway, namely the transport between the endoplasmic reticulum (ER) and Golgi apparatus, within the Golgi, from the Golgi to the plasma membrane (2, 3), and in autophagy-related vesicular fusion pathways (1, 4, 5, 6, 7, 8, 9, 10). Unlike most SNARE proteins, Ykt6 lacks a transmembrane domain and relies on reversible lipidation for recruitment to membranes to facilitate vesicular fusion. We and others have established that in the inactive state, Ykt6 is localized in the cytosol in a close conformation, whereby the regulatory longin domain is closely associated with the SNARE domain (1, 3, 10, 11, 12). In the active state, phosphorylation at the SNARE domain builds up the electrostatic potential which causes an intraconformational change separating the longin and the SNARE domain enabling reversible C-terminal lipid modifications (1, 11, 13, 14, 15, 16, 17). Lipidation allows Ykt6 to be anchored to the ER, Golgi, and plasma membranes (1, 13, 14, 16, 18, 19). Further, our laboratory also discovered that the subsequent dephosphorylation of the SNARE domain by CaN enhances SNARE–SNARE protein interactions, thereby facilitating vesicular fusion in both the secretory and autophagic pathways (1).

At excitatory synapses, α-amino-3-hydroxy-5-methyl-4-isoxazolepropionic acid receptors (AMPARs) modulate synaptic strength to facilitate information processing and storage (20, 21, 22, 23). GluA1 and GluA2 are the main AMPAR subunits that drive long-term potentiation (LTP) postdevelopment where they are rapidly inserted into synapses during LTP, a form of synaptic strengthening (24). While the roles of exocytosis and endocytosis in activity-dependent AMPAR transport at postsynaptic compartments have been well-established (25, 26, 27, 28, 29), little is known about the contribution of activity-dependent roles of the secretory pathway. GluA1 and GluA2 are good candidates for studying secretory transport in relation to synaptic plasticity in hippocampal neurons. First, GluA1 and GluA2, like the others of the AMPAR subunits, are synthesized in the ER where they assemble to form the channel (30, 31, 32). Second, GluA1A2 heterotetramers are the predominant AMPAR subtype that mediates synaptic transmission in the hippocampus (33, 34, 35). Third, GluA1 and GluA2 rely on local protein synthesis and activity-dependent trafficking for constant transport to the synaptic space (36, 37, 38). Lastly, synaptic insertion of GluA1 and GluA2-containing AMPARs is necessary for LTP (39, 40, 41, 42, 43, 44, 45, 46).

LTP triggers Ca^2+^ influx through both N-methyl-D-aspartic acid receptors (NMDARs) and AMPARs, and our previous findings in yeast and HeLa cells demonstrated that Ykt6 activity is regulated by CaN (1), whose activity is dependent on Ca^2+^. Moreover, we and others have implicated Ykt6 loss of function in α-synucleinopathies, neurodegenerative diseases characterized by deficits in LTP (1, 47, 48, 49). Therefore, we asked whether Ykt6, the master regulator of the secretory pathway, participates in delivering GluA1 and GluA2 subunits at synaptic compartments during LTP. Here, we show that Ykt6 is highly expressed in the hippocampus in the mammalian brain. Using primary pyramidal hippocampal neurons, we demonstrate that Ykt6 relocates to synaptic spines in response to LTP and promotes surface expression of GluA1 and GluA2. Moreover, we show that Ykt6 regulates spine morphology, the number of vesicles in the synaptic vesicular pools, and the amplitude and frequency of miniature excitatory postsynaptic currents. Taken together, our findings highlight a critical role for Ykt6 in hippocampal function and LTP with implications for α-synucleinopathies.

## Results

### Ykt6 is highly expressed in the mammalian hippocampus

**Figure 1:**
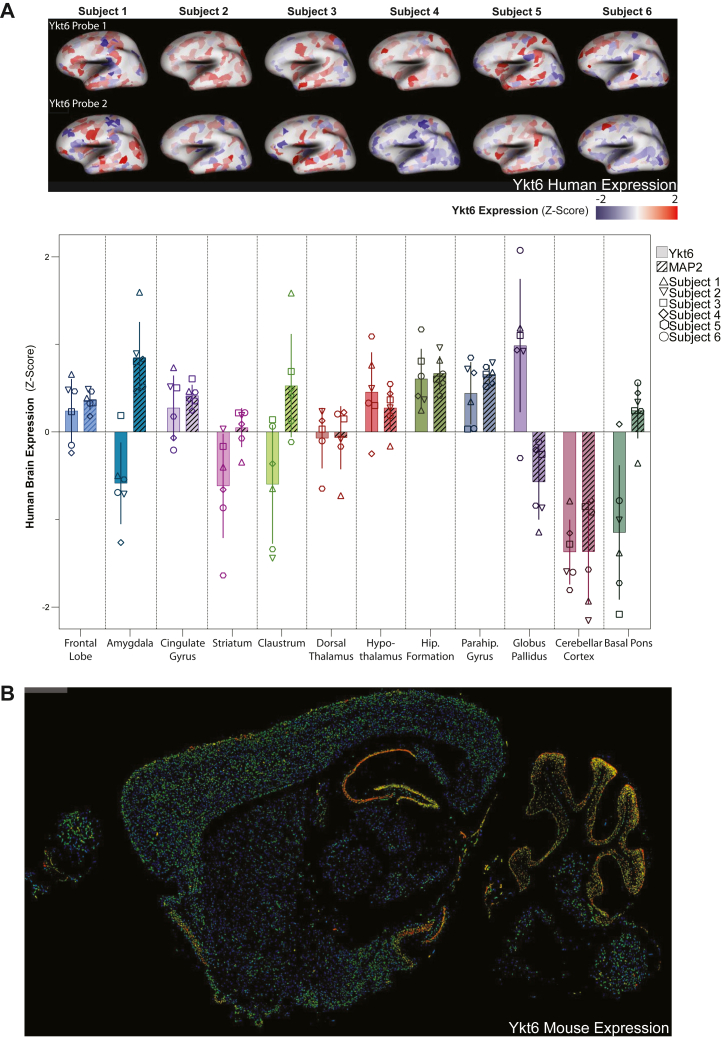
**Ykt6 is highly expressed in the hippocampus in the mammalian brain.***A*, *top:* sagittal view of Ykt6 expression by *in situ* hybridization assay and microarray with two Ykt6 probes in healthy adult human brains. Data obtained from the Allen Human Brain Atlas, https://human.brain-map.org/microarray/search/show?search_type=user_selections&user_selection_mode=2. *Bottom panel:* quantification of Ykt6 and microtubule-associated protein 2 (MAP2) from the indicated brain regions (153). N = 6 humans; MAP2 serves as a metric for high expression. Error bars represent SD. *B*, sagittal mouse brain section showing Ykt6 mRNA expression detected by fluorescence *in situ* hybridization. Data obtained from the Allen Mouse Brain Atlas, https://mouse.brain-map.org/experiment/show/71380453.

We first began by examining Ykt6 expression in the mammalian brain using the human and mouse brain atlas (https://portal.brain-map.org/atlases-and-data/bkp/abc-atlas) (50). In the human brain, data from six subjects who were otherwise healthy at the time of death was available. We examined Ykt6 expression using two different mRNA probes targeting Ykt6 in different brain regions, including the hippocampus. As a reference control, we analyzed the expression of the neuronal-specific microtubule-associated protein 2 (MAP2), a highly expressed neuronal protein. Relative to MAP2, the highest expression of Ykt6 was in the globus pallidus followed by the hippocampus (Fig. 1*A*, hippocampal formation). In the mouse brain, we examined Ykt6 expression using two different mRNA probes targeting Ykt6 in different brain regions, including the hippocampus. While the mRNA probe for Ykt6 was ubiquitously expressed throughout the brain, its expression was highest in the hippocampus and cerebellum, followed by the frontal cortex (Fig. 1*B*). Together, these data show that under physiological conditions Ykt6 is highly expressed in the mammalian hippocampus.

### Under resting conditions, Ykt6 is predominantly cytosolic, with partial colocalization at the Golgi apparatus and the ER within the soma and dendritic compartments of hippocampal neurons

**Figure 2:**
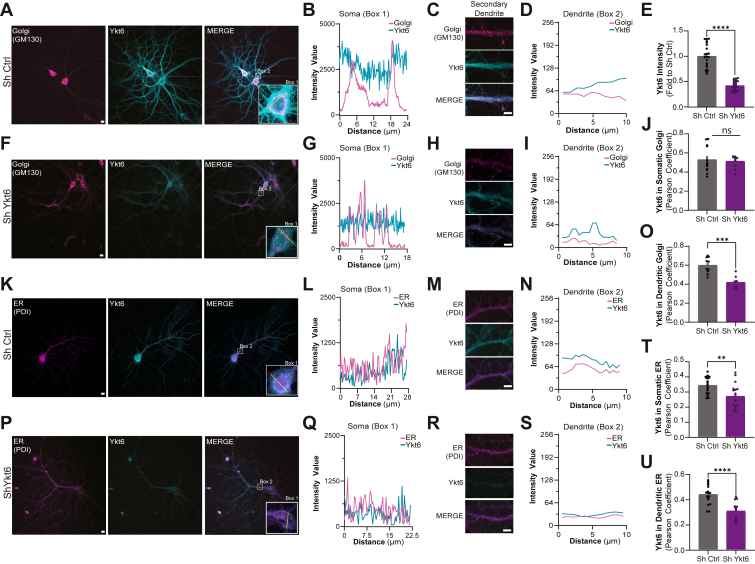
**Ykt6 is present in the cytosol, Golgi apparatus, and endoplasmic reticulum at somatic and dendritic locations in hippocampal pyramidal neurons.***A–J* and *O*, primary hippocampal pyramidal neurons were transduced with either ShRNA against Ykt6 (Sh Ykt6) or ShRNA scrambled sequence as control (Sh Ctrl) at DIV5, induced for Ykt6 knockdown with doxycycline starting at DIV8 and immunostained at DIV21 with GM130 (Golgi apparatus) and Ykt6. *A*, representative confocal immunofluorescence images of Golgi apparatus (*magenta*) and Ykt6 (*cyan*) for Sh Ctrl transduced neurons; the scale bar represents 10 μm. *B*, fluorescence intensity line scans of Golgi apparatus and Ykt6 from the soma (box 1) in (*A*). *C*, representative immunofluorescence image of secondary dendrites from (*A*). Golgi apparatus shown in *magenta*, Ykt6 in *cyan*; the scale bar represents 10 μm. *D*, fluorescence intensity line scans of the secondary dendrites from box 2 in (*A*). *E*, quantitation of Ykt6 fluorescence intensity from neurons transduced with Sh Ctrl or Sh Ykt6. *F*, representative confocal immunofluorescence images of Golgi apparatus (*magenta*) and Ykt6 (*cyan*) for Sh Ykt6 transduced neurons; the scale bar represents 10 μm. *G*, fluorescence intensity line scans of Golgi apparatus and Ykt6 from the soma (box 1) in (*F*). *H*, representative immunofluorescence image of secondary dendrites from (*F*). Golgi apparatus shown in *magenta*, Ykt6 in *cyan*; the scale bar represents 10 μm. *I*, fluorescence intensity linescans of the secondary dendrites from box 2 in (*F*). *J*, Pearson coefficient analysis for somatic Ykt6 and GM130 (Golgi apparatus) from neurons transduced with Sh Ctrl or Sh Ykt6. *K–N* and *P*–*U*, primary hippocampal pyramidal neurons were transduced with either ShRNA against Ykt6 (Sh Ykt6) or ShRNA scrambled sequence as control (Sh Ctrl) at DIV5, induced for Ykt6 knockdown with doxycycline starting at DIV8 and immunostained at DIV21 with protein disulfide isomerase (PDI, ER) and Ykt6. *K*, representative confocal immunofluorescence images of ER (*magenta*) and Ykt6 (*cyan*) for Sh Ctrl transduced neurons; the scale bar represents 10 μm. *L*, fluorescence intensity line scans of ER and Ykt6 from the soma (box 1) in (*K*). *M*, representative immunofluorescence image of secondary dendrites from (*K*). ER shown in *magenta*, Ykt6 in *cyan*; the scale bar represents 10 μm. *N*, fluorescence intensity line scans of the secondary dendrites from box 2 in (*K*). *O*, Pearson coefficient analysis for dendritic Ykt6 and GM130 (Golgi apparatus) from neurons transduced with Sh Ctrl or Sh Ykt6. *P*, representative confocal immunofluorescence images of ER (*magenta*) and Ykt6 (*cyan*) for Sh Ykt6 transduced neurons; the scale bar represents 10 μm. *Q*, fluorescence intensity line scans of ER and Ykt6 from the soma (box 1) in (*P*). *R*, representative immunofluorescence image of secondary dendrites from (*P*). ER shown in *magenta*, Ykt6 in *cyan*; the scale bar represents 10 μm. *S*, fluorescence intensity line scans of the secondary dendrites from box 2 in (*P*). *T*, Pearson coefficient analysis for somatic Ykt6 and ER from neurons transduced with Sh Ctrl or Sh Ykt6. *U*, Pearson coefficient analysis for dendritic Ykt6 and ER from neurons transduced with Sh Ctrl or Sh Ykt6. All stats, ∗∗*p* ≤ 0.01, ∗∗∗*p* ≤ 0.001, and ∗∗∗∗*p* ≤ 0.0001. N = 3, 3 to 7 cells per biological replicate. Error bars represent SEMs.

We next examined the intracellular localization of Ykt6 in rat primary hippocampal neurons in culture using immunofluorescence. In resting conditions, Ykt6 was primarily cytosolic in both somatic and dendritic regions (Fig. 2, *A*–*D*, *K*–*N*). Consistent with its role in the secretory pathway (51), Ykt6 also localized to the Golgi apparatus and to the ER, as shown by the colocalization with the Golgi marker GM130 and the ER-resident protein disulfide isomerase (Fig. 2, *A*–*D*, *J*–*O*, *T*, *U*). Ykt6 localization is specific, as reducing endogenous Ykt6 expression with a lentivirus expressing an inducible ShRNA targeting Ykt6 to knock down its expression (Sh Ykt6) significantly diminished Ykt6 detection compared to neurons expressing a scramble ShRNA sequence as a control (Sh Ctrl) (Fig. 2, *E*–*I*, *P*–*S*). Interestingly, while Ykt6 knockdown reduced its colocalization with the ER in both somatic and dendritic regions (Fig. 2, *T* and *U*), it selectively diminished Golgi colocalization in dendrites but not in the soma (Fig. 2, *J* and *O*). Together, these data indicate that under resting conditions, Ykt6 is predominantly present in the cytosol and can associate with the ER and Golgi apparatus at both somatic and dendritic sites.

### Ykt6 mobilizes to synaptic spines in a cLTP-dependent manner

**Figure 3:**
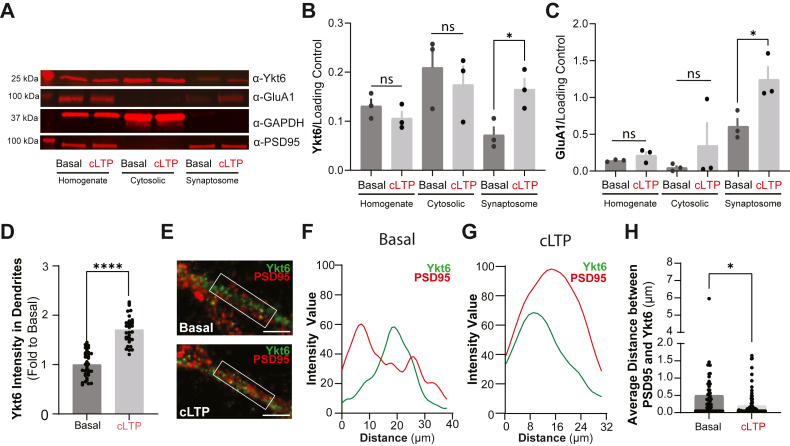
**Ykt6 mobilizes to the postsynaptic spines in a cLTP-dependent manner.***A*–*C*, adult rat brain tissue was exposed to glycine for chemical long-term potentiation (cLTP) or extracellular solution for basal condition (ECS), fractionated to cytosolic and synaptic fractions and immunoprobed for Ykt6, GluA1, GAPDH, and PSD95; GAPDH serves as a loading control for the homogenate and the cytosolic fractions, and PSD95 serves as a loading control for the synaptosomal fraction. Representative Western blot (*A*) and quantitation of Ykt6 (*B*) and GluA1 (*C*) levels over their respective loading controls. N = 3. *D*, quantification of Ykt6 fluorescence intensity in secondary dendrites of primary hippocampal pyramidal neurons treated with extracellular solution (ECS, basal condition) or glycine to induce chemical long-term potentiation (cLTP) at DIV21 and immunostained for Ykt6. N = 3. *E*–*H*, rat primary pyramidal hippocampal neurons were exposed to ECS for basal condition or glycine for cLTP at DIV21 and immunostained with the postsynaptic marker, postsynaptic density 95 (PSD95) and Ykt6. *E*, representative images of secondary dendrites from primary pyramidal hippocampal neurons exposed to extracellular solution (ECS) as basal condition (*top*) or glycine for cLTP (*bottom*). Ykt6 in *green* and PSD95 in *red*. The scale bar represents 5 μm. *F*, fluorescence intensity line scans from the region of interest for the basal condition (*white box* from *E*, *top*). *G*, fluorescence intensity line scans from the region of interest for the cLTP condition (*white box* from E, *bottom*). *H*, quantitation of average distance between PSD95 and Ykt6 from (*E*). Each data point represents aggregate average of all calculated shortest distances of each PSD95 puncta present in a selected region of interest from a secondary dendrite to the closest Ykt6 puncta. N = 2, 30 to 50 cells per biological replicate. All stats, unpaired *t* test, ∗*p* ≤ 0.05, ∗∗∗∗*p* ≤ 0.0001. Error bars represent SEMs.

We next asked whether the induction of chemical long-term potentiation (cLTP) using glycine, a widely employed method to enhance synaptic plasticity at postsynaptic spines (52, 53, 54, 55, 56, 57, 58), could modulate Ykt6 localization and thereby its activity at postsynaptic compartments. To address this, we first analyzed Ykt6 localization by isolating synaptosomal fractions through subcellular fractionation. These fractions contain portions of postsynaptic membranes, as evidenced by the presence of postsynaptic density protein 95 (PSD95), a well-established postsynaptic marker (Fig. 3*A*). Under basal/resting conditions, we detected Ykt6 at postsynaptic spines, as indicated by its presence in the PSD95 fraction (Fig. 3, *A* and *B*). As reported by many others (59), cLTP treatment increased GluA1 expression at postsynaptic spines by about 2-fold (Fig. 3, *A* and *C*). Importantly, cLTP induction also caused a significant increase in Ykt6 levels in the PSD95 fraction (Fig. 3, *A* and *B*). To corroborate this data, we analyzed Ykt6 presence at postsynaptic compartments by immunofluorescence. Consistent with the synaptosomal fraction data, cLTP treatment increased Ykt6 localization at postsynaptic compartments, as evidenced by its enhanced intracellular localization in dendritic compartments (Fig. 3*D*) and increased proximity to PSD95 (Fig. 3, *E*–*H* and S1*A*). Together, these data indicate that while Ykt6 is present at postsynaptic spines under resting conditions, it is further mobilized to these sites upon cLTP induction.

### Ykt6 regulates GluA1 and GluA2 expression at synaptic spines in a cLTP-dependent manner

**Figure 4:**
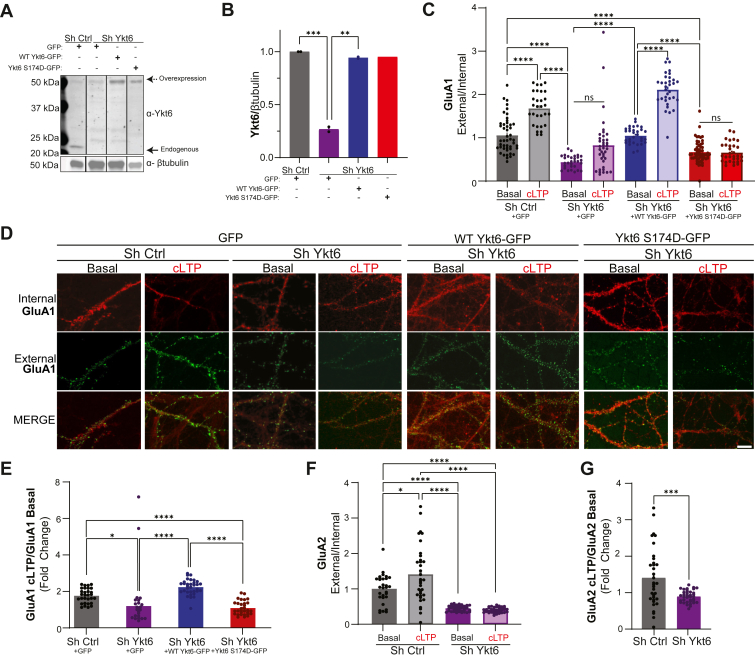
**Ykt6 regulates GluA1 and GluA2 localization at synaptic spines in a cLTP-dependent manner.***A*, representative Western blot for Ykt6 expression from rat primary hippocampal neurons cotransduced with four different conditions: 1) Sh Ctrl + GFP, 2) Sh Ykt6 + GFP, 3) Sh Ykt6 + WT Ykt6-GFP, and 4) Sh Ykt6 + Ykt6 S174D-GFP. *Solid arrow*, endogenous Ykt6; *dashed arrow*, exogenous Ykt6. β-Tubulin serves as a loading control. *B*, quantification of Ykt6 endogenous expression over loading control from (*A*). N = 2. One-way ANOVA with Welch’s correction and Dunnett’s multiple comparisons test. F(2.000, 1.212) = 840.8, *p* = 0.0125; *p* value for *post hoc* multiple comparisons test, for Sh Ctrl + GFP, *versus* Sh Ykt6 + GFP, 0.0010; Sh Ykt6 + GFP *versus* Sh Ykt6 + WT Ykt6-GFP, 0.0013; otherwise nonsignificant. *C*–*D*, rat primary hippocampal neurons from (*A*) were exposed to extracellular solution (ECS) or glycine for chemical long-term potentiation (cLTP) at DIV21 and immunolabeled for external and internal GluA1. N = 3, 8 to 10 cells per biological replicate. *C*, ratios of external to internal GluA1 levels normalized to the control (basal condition, cotransduced with Sh Ctrl and GFP construct). One-way ANOVA with Welch’s correction and Dunnett’s multiple comparisons test. F(18.00, 36.44) = 16.53, *p* < 0.0001. All *p* values for *post hoc* multiple comparisons test < 0.0001. *D*, representative immunofluorescence images of the secondary dendrites from (*C*). Internal GluA1 in *red* and external GluA1 in *green*. The scale bar represents 10 μm. *E*, fold change in external and internal GluA1 ratios between basal and cLTP conditions. One-way ANOVA with Welch’s correction and Dunnett’s multiple comparisons test. *F*–*G*, rat primary hippocampal neurons were cotransduced with two different conditions: 1) Sh Ctrl or 2) Sh Ykt6 and were exposed to ECS or glycine for cLTP at DIV21 and immunolabeled for external and internal GluA2. N = 3, 8 to 10 cells per biological replicate. *F*, ratios of external to internal GluA1 levels normalized to the control (basal condition, transduced with Sh Ctrl). One-way ANOVA with Welch’s correction and Dunnett’s multiple comparisons test. F(3.000, 50.46) = 40.29, *p* < 0.0001; *p* values for *post hoc* multiple comparisons test < 0.0001 except for Sh Ctrl (basal *versus* cLTP) = 0.0464. *G*, fold change in external and internal GluA2 ratios between basal and cLTP conditions. Unpaired *t* test with Welch’s correction. All stats, ∗*p* ≤ 0.05, ∗∗*p* ≤ 0.01, ∗∗∗*p* ≤ 0.001, and ∗∗∗∗*p* ≤ 0.0001. Error bars represent SEMs.

Recruitment of Ykt6 to postsynaptic compartments in a cLTP-dependent manner suggested its contribution to synaptic strengthening and plasticity. Synaptic plasticity is mediated by the synaptic distribution of AMPARs (20, 21, 22, 23). AMPAR subunit assembly occurs within the ER (31, 32, 60, 61). Therefore, Ykt6-mediated ER transport of AMPARs may modulate the synaptic pool available for membrane insertion, thereby impacting synaptic plasticity. Among the four AMPARs subunits (GluA1-4), GluA1, and GluA2 are good candidates for studying secretory transport in relation to synaptic plasticity in hippocampal neuron given that synaptic transmission is primarily mediated by GluA1A2 heterotetramers (33, 34). While GluA2 remains relatively stable near the plasma membrane, GluA1 depends on continuous intracellular trafficking to reach the synaptic space. Moreover, the synaptic insertion of GluA1-containing AMPARs is necessary for LTP (33, 39, 40, 41, 42, 43, 44, 45, 46, 57, 62). To determine whether Ykt6 influences the distribution of endogenous AMPARs, we employed two complementary approaches: an antibody feeding assay and a cell surface biotinylation assay. Antibody feeding assays are commonly employed in the field (59, 63, 64, 65, 66, 67) and quantify surface AMPARs relative to total by comparing external and internal fractions, thereby providing a measure of surface expression rather than overall AMPAR levels. Cell surface biotinylation assays selectively label membrane bound proteins by using the membrane impermeable biotin reagent, which after lysis can be isolated by using streptavidin-coated beads, which bind biotin with high affinity (68). For both assays, rat primary hippocampal neurons were cotransduced with: 1) lentiviruses expressing an inducible ShRNA targeting Ykt6 to knock down its expression (hereafter referred to as Ykt6 knockdown), or a scramble ShRNA sequence as a control (hereafter referred to as control), and 2) either an N terminally GFP-tagged WT human Ykt6 (WT Ykt6-GFP), a Ykt6 SNARE-deficient mutant (S174D Ykt6-GFP) (1) both resistant to the Ykt6 ShRNA to rescue Ykt6 expression to endogenous levels, or GFP alone as control. Western blot analysis confirmed that Ykt6 knockdown reduces endogenous Ykt6 expression by approximately 70% and that the WT and S174D Ykt6-GFP–resistant constructs successfully restore Ykt6 expression to endogenous levels (Fig. 4, *A*, *B* and S2).

Under resting conditions, Ykt6 knockdown slightly reduced surface expression of GluA1 compared to the control condition by antibody feeding assay as well as by cell surface biotinylation (Fig. 4, *C*, *D*, S1, *B* and *C*). The effect on GluA1 surface levels under basal conditions was specific to Ykt6, as coexpression of the WT Ykt6 knockdown-resistant construct restored basal GluA1 surface levels (Fig. 4, *C*, *D*, S1, *B* and *C*). Importantly, GluA1 surface expression was dependent on Ykt6’s vesicular fusion activity, as the S174D Ykt6 mutant, which is unable to pair with SNAREs and is therefore fusion-deficient, failed to rescue basal GluA1 surface levels (Fig. 4, *C* and *D*). As previously reported (59), cLTP treatment led to a 2-fold increase in GluA1 surface expression under control conditions by antibody feeding assay as well as by cell surface biotinylation (Fig. 4, *C* and *D*, gray bars and S1, *B* and *C*). In contrast, Ykt6 knockdown prevented the cLTP-induced increase in GluA1 surface expression (Fig. 4, *C* and *D*, purple bars and S1, *B* and *C*). This effect was specific to the SNARE function of Ykt6, as re-expression of WT Ykt6 restored the 2-fold increase in GluA1 surface levels following cLTP treatment (similar to control conditions) (Fig. 4, *C* and *D*, blue bars and S1, *B* and *C*), whereas the S174D mutant failed to do so (Fig. 4, *C* and *D*, red bars). To distinguish the basal *versus* activity-dependent contribution of Ykt6 to GluA1 surface expression, we analyzed the cLTP data relative to basal levels (Fig. 4*E*). Relative to control conditions, rescue with WT Ykt6, but not the SNARE-deficient S174D Ykt6 mutant, restored the upregulation of GluA1 surface levels upon cLTP treatment (Fig. 4*E*).

GluA1A2 heteromers are the predominant AMPAR type that mediates synaptic transmission in the hippocampus (33, 34, 35). GluA1A2 heteromers are the predominant AMPAR subtype that mediates synaptic transmission in the hippocampus (22, 33, 34, 35, 69, 70), and both GluA1 and GluA2 subunits are regulated by the secretory pathway (71, 72, 73, 74). We therefore examined by subcellular fractionation, antibody feeding assays, as well as by cell surface biotinylation, if endogenous GluA2 surface levels were also regulated by Ykt6. As previously reported (59), cLTP treatment led to a slight increase in GluA2 surface expression reflected by an increase in GluA2 levels in postsynaptic synaptosomal fractions, by antibody feeding assay, as well as by cell surface biotinylation (Fig. 4, *F* and *G*, gray bars and S1, *D*–*H*). In contrast, Ykt6 knockdown prevented the increase in GluA2 surface expression upon cLTP (Fig. 4, *F* and *G*, purple bars and S1, *D*–*H*). The effect on GluA2 surface levels under both basal and cLTP-induced conditions was specific to Ykt6, as coexpression of a knockdown-resistant WT Ykt6 construct restored basal GluA2 surface levels, as measured by cell surface biotinylation (S1, *F* and *G*). Together, these data indicate that while Ykt6 has only a modest effect on GluA1 and GluA2 surface levels under basal conditions, it plays a key role in the activity-dependent regulation of GluA1 and GluA2 surface expression, as evidenced by the pronounced increase observed following cLTP treatment.

### Ykt6 modulates both presynaptic and postsynaptic compartments of glutamatergic neurotransmission

**Figure 5:**
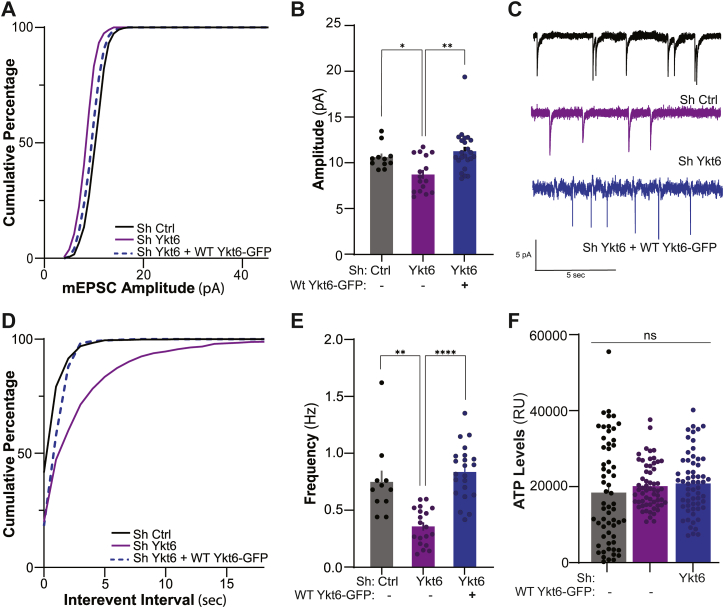
**Ykt6 modulates presynaptic and postsynaptic compartments of glutamatergic neurotransmission.***A*–*E*, rat primary hippocampal neurons were cotransduced with three different conditions: 1) Sh Ctrl (*black*), 2) Sh Ykt6 (*purple*), or 3) Sh Ykt6 + WT Ykt6-GFP (*blue*), and patch-clamped in whole-cell configuration at DIV 18 to 21 in the presence of tetrodotoxin (TTX), D-2-amino-5-phosphonovalerate (D-APV), and picrotoxin (PTX) to isolate AMPA currents (*A–E*). *A*, cumulative distribution of the amplitudes for all events per condition. *B*, average amplitude per condition. F(2, 46.31) = 9.053, *p* = 0.0005. *p* value for *post hoc* multiple comparisons test, for control *versus* Ykt6 knockdown = 0.0162; Ykt6 knockdown *versus* rescue = 0.0022. *C*, representative traces of cultures in (*A*); N = 3, 4 to 8 cells per biological replicate. *D*, cumulative distribution of time between each event for every event per condition (*p* < 0.0001, Kolmogorov-Smirnov test with Bonferroni correction). *E*, average frequency of events for each condition. F(2, 23.18) = 30.66, *p* < 0.0001. *p* value for *post hoc* multiple comparisons test, for control *versus* Ykt6 knockdown = 0.0075; Ykt6 knockdown *versus* rescue < 0.0001. *F*, quantitation of endogenous ATP levels at DIV21 from cultures in (*A*). N = 3. All stats, one-way ANOVA with Welch’s correction and Dunnett’s multiple comparisons test. ∗*p* ≤ 0.05, ∗∗*p* ≤ 0.01, and ∗∗∗∗*p* ≤ 0.0001. Error bars represent SEMs.

Expression of AMPARs at postsynaptic membranes regulates the amplitude of excitatory postsynaptic currents. We therefore asked if Ykt6 modulation of surface expression of GluA1 and GluA2 at resting conditions could have a role on miniature excitatory postsynaptic currents (mEPSCs). Rat primary pyramidal hippocampal neurons transduced with Sh Ctrl, Sh Ykt6, or Sh Ykt6, and WT Ykt6-GFP were patched and recorded in the presence of NMDA receptor blocker D-2-amino-5-phosphonopentanoic acid, voltage-gated sodium channel blocker tetrodotoxin (TTX), and gamma-aminobutyric acid subunit A receptor antagonist picrotoxin (PTX) to isolate AMPA-mediated currents. Ykt6 knockdown reduced the amplitude of mEPSCs compared to the control without significantly changing the distribution of the mEPSC amplitudes (Fig. 5, *A*–*C*). This effect is specific to Ykt6 as coexpression with the WT Ykt6 knockdown-resistant construct (rescue) restored the amplitude (Fig. 5, *A* and *B*; control: 10.64 ± 0.400pA, Ykt6 knockdown: 8.728 ± 0.486pA, rescue: 11.25 ± 0.482pA, Welch’s ANOVA, F (2, 46.31) = 9.053, *p* = 0.0005). Pairwise comparisons using Dunnett's T3 multiple comparisons test indicated that Ykt6 knockdown had significantly lower amplitudes than the control (*p* = 0.0162) or the rescue (*p* = 0.0022) (Fig. 5, *A*–*C*). The observed reduction in mEPSC amplitude aligns with the decrease in GluA1 and GluA2 surface density at the postsynaptic sites that we observed by antibody feeding assays and cell surface biotinylation (Fig. 4, *C*–*G*, S1, *B* and *C*, *F*–*H*). Moreover, we detected a concordant decrease in the inter-event cumulative distribution (Fig. 5, *C* and *D*; control *versus* Ykt6 knockdown, *D =* 0.595, *p <* 0.0001; Ykt6 knockdown *versus* rescue, *D* = 0.7907, *p* < 0.0001) and the frequency of inter-event intervals in Ykt6 knockdown compared to the control and rescue (control: 0.750 ± 0.010 Hz, Ykt6 knockdown: 0.357 ± 0.037 Hz, rescue: 0.8362 ± 0.051 Hz, Welch’s ANOVA, F(2, 23.18) = 30.66, *p* < 0.0001) (Fig. 5, *C* and *E*). Pairwise comparisons using Dunnett's T3 multiple comparisons test indicated that Ykt6 knockdown had significantly lower amplitudes than the control (*p* = 0.0075) or the rescue (*p* < 0.0001). Importantly, the decrease in mEPSCs is not due to neuronal viability issues, as ATP levels—a reliable indicator of cell viability—remained unaffected by Ykt6 knockdown (Fig. 5*F*). Together, these results indicate that Ykt6 modulates presynaptic and postsynaptic electrophysiological dynamics.

### Ykt6 regulates spine and dendritic morphology

**Figure 6:**
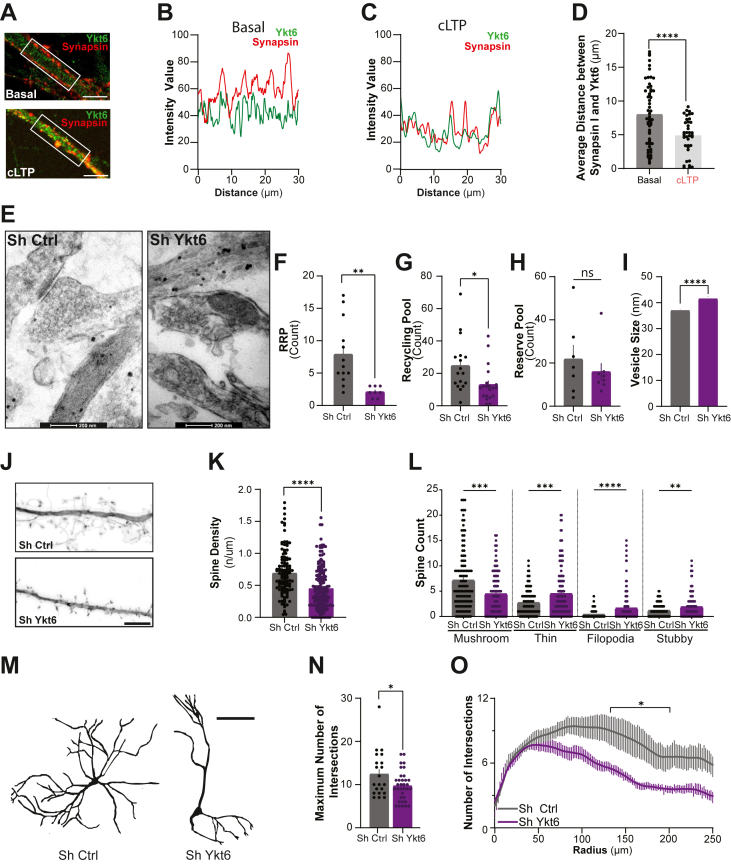
**Ykt6 modulates neuronal structures related to synaptic plasticity.***A*–*D*, rat primary pyramidal hippocampal neurons exposed to extracellular solution (ECS) for basal condition or glycine for chemical long-term potentiation (cLTP) at DIV21 and immunostained with the presynaptic marker, synapsin I, and Ykt6. *A*, representative images of secondary dendrites under basal condition (*top*) or cLTP (*bottom*). Ykt6 in *green* and synapsin I in *red*. The scale bar represents 5 μm. *B*, fluorescence intensity line scans from the region of interest for the basal condition (*white box* from A, *top*). *C*, fluorescence intensity line scans from the region of interest for cLTP condition (*white box* from A, *bottom*). *D*, quantitation of average distance between synapsin I and Ykt6 from (*A*). Each data point represents aggregate average of all calculated shortest distances of each synapsin I puncta present in a selected ROI from a secondary dendrite to the closest Ykt6 puncta. N = 2, 30 to 50 cells per biological replicate. Unpaired *t* test. *E*–*I*, rat primary hippocampal pyramidal neurons were transduced with either Sh Ctrl or Sh Ykt6 and processed for electron microscopy imaging at DIV21. *E*, representative EM images of dendritic spines from the hippocampal cultures described above. *F*, synaptic vesicle count for readily releasable pool (RRP). Each point is count for one image from (*E*). *G*, synaptic vesicle count for recycling pool from (*E*). *H*, synaptic vesicle count for reserve pool from (*E*). *I*, average synaptic vesicle size from (*E*). *J*–*L*, rat primary hippocampal pyramidal neurons were transduced with either Sh Ctrl or Sh Ykt6, transfected with mScarlet on DIV19, and imaged using confocal microscopy on DIV21. *J*, representative confocal immunofluorescence images of secondary dendrites and dendritic spines. The scale bar represents 2 μm. *K*, spine density (number of spines per μm) from (*J*). *L*, quantitation of the number of spines per each categorical spine type for each condition. Each data point represents one secondary dendrite. Unpaired *t* test with Welch’s correction. N = 2 biological replicates, 20 to 30 cells per biological replicate. *M*–*O*, rat primary hippocampal neurons were transduced with either Sh Control or Sh Ykt6, transfected with mCherry on DIV19, and analyzed at DIV21 using Sholl analysis. *M*, representative skeletonized images from pyramidal hippocampal neurons. *N*, quantitation of maximum number of intersections for hippocampal pyramidal neurons from (*M*). Unpaired *t* test with Welch’s correction. *O*, Sholl analysis of hippocampal pyramidal neurons. Two-way ANOVA with mixed-effects model and multiple comparisons test. Radius F(130, 5908) = 20.09, *p* < 0.0001; Sh Ctrl *versus* Sh Ykt6 F(1, 53) = 9.711, *p* = 0.0030; Radius × Sh Ctrl *versus* Sh Ykt6 F(130, 5908) = 3.445, *p* < 0.0001. N = 20 to 30, 10 to 15 cells per biological replicate. The scale bar represents 100 μm. All stats, ∗*p* ≤ 0.05, ∗∗*p* ≤ 0.01, ∗∗∗*p* ≤ 0.001, and ∗∗∗∗*p* ≤ 0.0001. Error bars represent SEMs.

The reduction in mEPSC amplitude following Ykt6 loss suggested a potential presynaptic role for Ykt6. To explore this, we first assessed Ykt6 localization in presynaptic compartments using immunofluorescence. Ykt6 was detected in presynaptic regions under basal conditions, and its presence increased following cLTP induction, as indicated by enhanced colocalization with synapsin 1, a well-established presynaptic marker (Fig. 6, *A*–*D* and S1*I*). To investigate whether the changes in synaptic transmission were due to changes in the number of presynaptic vesicles associated with the synapse, we performed EM on rat primary pyramidal hippocampal neurons transduced with Sh Ctrl or Sh Ykt6. In line with previous studies (75, 76), we classified the vesicles that are touching synaptic contact points as the readily releasable pool (RRP), those proximal to the contact point but not touching membranes as recycling pool, and those distal from the contact point as reserve pool. Ykt6 knockdown reduced synaptic vesicles in both the RRP and the reserve pool, while the distal pool remained unaffected (Fig. 6, *E*–*H*). Additionally, we observed a slight increase in the average size of synaptic vesicles across all three pools in the Ykt6 knockdown condition (Fig. 6*I*).

The EM images showed immature filopodia-like morphology of the spine under Ykt6 knockdown, whereas more mature mushroom-like shape spines were present in the control (Fig. 6*E*), in line with previous studies establishing a correlation between synaptogenesis and spine morphology (58, 77, 78). We therefore investigated whether Ykt6 regulation of excitatory neuronal activity could modulate spine density and morphology, hallmarks of LTP. Rat primary pyramidal hippocampal neurons transduced with Sh Ctrl or Sh Ykt6 were transfected with mScarlet at days *in vitro* (DIV) 19 and analyzed at DIV21 for spine visualization. Ykt6 knockdown reduced the overall number of spines/micrometer compared to control (Fig. 6, *J* and *K*). Moreover, Ykt6 knockdown reduced the number of mature mushroom-like spines and increased the immature thin, filopodia, stubby-like spines (Fig. 6*L*).

The secretory pathway is not only important for neurotransmitter release and synapse morphology but it also provides proteins essential for dendritic morphogenesis. Dendritic arborization is crucial not only during neurodevelopment but also in adult neurons, where synaptic activity can influence the morphology of the dendritic arbor (39, 46, 79, 80, 81, 82, 83, 84). To investigate whether Ykt6’s role in the secretory pathway affects dendritic morphology, hippocampal primary neurons transduced with either Sh Ykt6 or Sh Ctrl were transfected with mCherry to visualize the dendritic arbor and analyzed at DIV21 using Sholl analysis to assess branching complexity (85). Ykt6 knockdown reduced dendritic arborization compared to control, particularly in the regions distant from the soma (Fig. 6, *M*–*O*). Collectively, these data indicate that Ykt6 is present at presynaptic compartments where it regulates vesicular pools, spine and dendritic arbor morphology, and critical features of synaptic plasticity.

## Discussion

Ykt6's role in vesicular trafficking has been well studied in yeast and mammalian systems, yet its physiological function in the brain remains largely unknown. Here, we show that Ykt6 is highly expressed in the mammalian hippocampus. In hippocampal pyramidal neurons, Ykt6 is associated with the secretory organelles ER and Golgi (86) and can be mobilized to synaptic compartments upon cLTP induction. Furthermore, we demonstrate that Ykt6 regulates both basal and cLTP-dependent GluA1 and GluA2 surface levels. Following development, most GluA1-containing AMPARs exist as GluA1/GluA2 heterotetramers (38, 41, 87, 88, 89). However, whether GluA1 homotetramers or GluA1/GluA2 heterotetramers are essential for LTP remains unresolved (20, 22, 63, 66, 69). Our findings show that Ykt6 knockdown affects both GluA1 and GluA2 expression, albeit to different extents, supports the idea that both receptor subtypes contribute to LTP. Moreover, Ykt6 modulates the availability of vesicular pools at the synapse and ultimately impacting mEPSCs, synapse structure, and dendritic morphology. Reductions in GluA1 and GluA2 expression may result from the presence of immature spines (90), which are also observed with the loss of Ykt6. Downregulation of Ykt6 slightly increases vesicle size, but disrupts GluA1 and GluA2 trafficking to the surface, reducing synaptic amplitude and also contributing to longer inter-event intervals. These prolonged intervals have been linked to impairments in the RRP (91) and may also result from decreased spine density and increased presence of immature spines (92, 93)—all of which we observe under Ykt6 deficiency.

The Ykt6 SNARE-deficient mutant (S174D Ykt6-GFP) provided an invaluable tool to dissect the SNARE-mediated contribution of Ykt6 to GluA1 and GluA2 surface trafficking. While Ykt6 is an essential R-SNARE protein with established roles not only in the secretory pathway, but in the exocytosis and autophagy pathways (1, 4, 5, 6, 7, 8, 10, 14, 15, 19, 94, 95, 96, 97, 98, 99, 100), our findings suggest a novel role for Ykt6 in regulating AMPARs *via* the secretory pathway, distinct from its roles in autophagy or exocytosis. This hypothesis is supported by the following evidence. While surface expression of AMPARs can be modulated by autophagy in an activity-dependent manner (86, 101, 102, 103, 104, 105), autophagy primarily facilitates the degradation of these receptors. In contrast, our data demonstrate that Ykt6 has a positive role in both basal and cLTP-dependent AMPAR trafficking, enhancing receptor surface expression rather than contributing to their degradation.

The R-SNARE VAMP2 (synaptobrevin-2) plays a crucial role in forming well-established SNARE complexes, such as SNAP25–syntaxin-1A/B–VAMP2 or VAMP2–syntaxin-3–SNAP-47, which are essential for AMPAR exocytosis and endocytosis at postsynaptic compartments (106, 107, 108, 109, 110, 111, 112). Although Ykt6, another R-SNARE, could theoretically substitute for VAMP2 in these complexes, it remains unclear how Ykt6 might displace VAMP2 from its known binding partners. This suggests that Ykt6 is unlikely to regulate GluA1 and GluA2 surface expression *via* exocytosis.

Neurons have evolved specialized ER and Golgi outposts at distal locations from the soma to enable rapid and precise delivery of protein cargo to synapses (113, 114). Extensive networks of smooth ER tubules and sheets in dendrites and spines suggest local secretory trafficking, bypassing traditional somatic secretory pathways (28, 73, 82, 115, 116, 117, 118, 119). ER exit sites and COPII subunits have been observed at both proximal and distal dendritic locations in hippocampal neurons (71, 116, 120). Similarly, Golgi outposts in dendritic and axonal compartments support localized secretory functions essential for synaptic plasticity and neuronal responses. Local AMPAR synthesis in dendrites has been observed to rely on the ER-Golgi intermediate compartment (121, 122, 123). Moreover, recent evidence suggests alternative sources of AMPAR synthesis coupled to synaptic plasticity-dependent signals (22, 124, 125). For example, in *Drosophila*, intra-Golgi transport is regulated by Ca^2+^-calmodulin–dependent kinase II, an enzyme activated by LTP (126, 127, 128, 129). Additionally, the dendritic ER plays a critical role in long-term depression (LTD), a form of synaptic plasticity. During LTD, ER Ca^2+^ release is enhanced, NMDAR trafficking is upregulated, and the ER network near spines becomes more complex (130, 131, 132, 133). Moreover, recent evidence suggests alternative sources of AMPAR synthesis coupled to synaptic plasticity-dependent signals (22, 124, 125). The fact that Ykt6 is present at ER and Golgi outposts in secondary dendrites under basal conditions, relocalizes to synaptic compartments upon cLTP induction, and suggests a potential role of Ykt6 in distal secretory outposts. Understanding how Ykt6 regulates AMPAR trafficking at distal secretory outposts will be crucial for elucidating its molecular mechanisms and functional significance, particularly in the context of neuronal plasticity and synaptic function. Furthermore, a more thorough understanding of whether Ykt6 affects AMPAR exocytosis, recycling, and/or lateral diffusion is critical to further appreciate the contribution of Ykt6 to AMPAR dynamics.

Misfolding of α-synuclein leads to a group of neurodegenerative diseases collectively known as synucleinopathies, which includes dementia with Lewy bodies and Parkinson’s disease dementia (134, 135, 136, 137, 138, 139). A block in ER-to-Golgi transport and a loss of Ykt6 function is a hallmark of α-synuclein pathology across various model systems (1, 4, 5, 17, 140, 141). Previous findings from our laboratory demonstrated that α-synuclein causes ER-to-Golgi trafficking deficits. This in turn, leads to a pathological increase in cytosolic Ca^2+^ levels and constitutive activation of the Ca^2+^-dependent phosphatase CaN, which results in Ykt6 constitutive dephosphorylation and loss of function (1). Moreover, other groups have shown that α-synuclein disrupts AMPAR and NMDAR surface levels at synapses, impairing LTP and spatial learning (47, 49, 142, 143, 144, 145). Consistent with these findings, here, we have shown that physiological Ykt6 loss of function in the hippocampus results in defects in both basal and cLTP-dependent GluA1 and GluA2 surface expression. Therefore, our data can provide a mechanistic explanation for how α-synuclein disrupts AMPAR trafficking and synaptic plasticity. Furthermore, dysregulated surface expression of AMPAR has been implicated not only in Parkinson’s disease but also in other neurodegenerative disorders associated with cognitive and memory deficits, such as Alzheimer’s disease (88, 89, 124, 146, 147, 148, 149, 150, 151, 152, 153, 154). Thus, our discoveries of a novel role for Ykt6 in AMPAR regulation may provide new therapeutic targets for treating common neurodegenerative disorders.

## Experimental procedures

### Quantification of Ykt6 expression in human and mice brains

The data for both human and mice brains were obtained from the Allen Brain Atlas (https://human.brain-map.org/microarray/search/show?search_type=user_selections&user_selection_mode=2). The data was generated by the Allen Institute using two mRNA probes (A_23_P134527 and A_24_P372048) for *in situ* hybridization and microarray assays from six human subjects (155). The Z-score of all six subjects were averaged and compared against the Z-score of MAP2 using two mRNA probes (A_23_P153917 and A_24_P231483) for each brain region.

### Primary hippocampal cultures

Embryonic rat hippocampal neurons were isolated from euthanized pregnant Sprague–Dawley rats at embryonic day 18 using a modified protocol from Lesuisse and Martin. Protocol was approved by Northwestern University administrative panel on laboratory animal care. Embryos were harvested by Caesarean section and hippocampi were isolated and dissociated with 0.25% trypsin without EDTA (Invitrogen, 15090-046) digestion for 15 min at 37 °C and trituration with 1 ml plastic tip. Poly-D-lysine (Sigma, P-1149)-coated 1.5 coverslips (Neuvitro, GG-12--1.5-oz) in 24-well plates were seeded with 5 × 10^5^ cells accordingly in neurobasal medium (Gibco, 21103-049) supplemented with 10% heat-inactivated fetal bovine serum (Gibco), 0.5 mM glutamine (Gibco), penicillin (100 IU/ml), and streptomycin (100 μg/ml) (Gibco). Before seeding, cells were counted using the automated cell counter TC10 (Bio-Rad) and viability (90–95%) was checked with Trypan blue stain (0.4%, Gibco 15250-061). After 1 h incubation at 37 °C, media were changed to neurobasal medium (Gibco, 21103-049) supplemented with B27 (Gibco, 17504-044) and 0.5 mM glutamine. One half (out of 500 μl volume for 24-well plates) of the media were changed on DIVs 5, 9, 12, 16, and 19.

### Plasmids

Nontargeting ShRNA (Horizon Discovery Dharmacon, VSC11656) or ShRNA targeting Ykt6 (Horizon Discovery Dharmacon, V3SH7669) were expressed in mammalian cells using lentiviral induction at DIV5.

Expression was verified visually by the expression of turboRFP.

Human eGFP-Ykt6 (a kind gift from Dr Joseph Mazzulli, Northwestern University) and eGFP were expressed in mammalian cells using lentiviral induction at DIV5 at half the multiplicity of infection used with ShRNAs.

Human eGFP-Ykt6 was mutated using Q5 Site-Directed Mutagenesis Kit with the following primers: Forward 5′GGTGTCCAAAGACGAGGTGCTGG-3′ and Reverse 5′-AAGTCATCTAGCTTCTCAC-3′ sequences to generate the S174>D substitution.

### Doxycycline treatment of cultures

Primary hippocampal cultures that were infected with either nontargeting ShRNA or ShRNA targeting Ykt6 were treated with doxycycline. Targeted silencing of Ykt6 in primary hippocampal cultures was induced using doxycycline treatments at a concentration of 1 nM at DIV 8, 12, 15, and 19 before used for experiments. Viral multiplicity of infection and doxycycline titrations were performed on new batches before applied experimentally and confirmation of knockdown and expression of turbo RFP was regulated.

### Chemical LTP induction

The samples were washed once with warmed extracellular solution (ECS) containing (in mM): 150 NaCl, 2 CaCl_2_, 5 KCl, 10 Hepes, 30 glucose, 0.001 TTX, 0.01 strychnine, and 0.03 PTX at pH 7.4, then exposed to 300 μM glycine in ECS for 3 min (external/internal staining) or 6 min (Western blot) at room temperature (RT), then washed with ECS thrice, and then incubated at 37 °C for 20 min prior to further processing.

### External/internal staining

External and internal glutamatergic receptors were stained according to the protocol by Chiu *et al.* (59). Coverslips with primary hippocampal cultures were transferred to parafilm-covered dishes and incubated in primary antibody (GluA1: Invitrogen, MA5-27694; GluA2: Sigma-Aldrich, MAB397) at the concentration of 1:100 diluted in conditioned media for 15 min at RT and then washed three times with warm conditioned media. The coverslips were then washed once with PBS supplemented with 1 mM MgCl_2_ and 0.1 mM CaCl_2_ (PBS+). The cells were then fixed by incubating with fresh 4% paraformaldehyde (PFA) and 4% sucrose in PBS for 8 min. The cells were then washed three times with PBS and then blocked with 10% normal goat serum (NGS) in PBS for minimum 30 min at RT. The cells were then incubated with 1:400 dilution of goat anti-mouse Alexa Fluor 405 secondary antibody (Invitrogen, A-31553) or goat anti-rabbit Alexa Fluor 405 secondary antibody (abcam, ab175652) in 3% NGS in PBS for 1 h at RT. The cells were then washed 3 times with PBS.

For internal staining, the cells were then permeabilized with 0.25% Triton X-100 (Thermo Fisher Chemicals, A16046.AP) for 7 min at RT and then blocked with 10% NGS in PBS for minimum 30 min at RT. The intracellular receptors were then labeled by incubating the cells with the same primary antibodies at the same concentration, and anti-MAP2 chicken antibody (abcam, ab318993) at 1:10,000 dilution, but diluted in 3% NGS in PBS overnight in 4 °C. The next morning, cells were washed 3 times with PBS, then labeled with goat anti-mouse Alexa Fluor 647 secondary antibody (Invitrogen, A-21235) or goat anti-rabbit Alexa Fluor 405 secondary antibody (Invitrogen, A-21245) at 1:1000 dilution and goat anti-chicken Alexa Fluor 488 secondary antibody (Invitrogen, A-11039) at 1:400 dilution in 3% NGS in PBS for 1 h at RT. The cells were then washed 3 times with PBS and once with deionized water and then mounted using Prolong Gold antifade mountant (Invitrogen, P36930). The cells were imaged using Nikon A1R GaAsP point-scanning laser confocal microscope using 63× oil-immersion objective (NA = 1.4) with z-series of 10 to 20 images, taken at 0.3 μm intervals, with 2048 × 2048 pixel resolution. Only cells positive for MAP2 were acquired. The external to internal expression ratio was calculated for the basal condition with GFP and Sh Ctrl, or Sh Ctrl only. This value was then normalized to 1, and all other ratios normalized to the basal condition.

### Ykt6 staining

Primary hippocampal cultures as described above at DIV21 were washed once with PBS+ and then fixed with fresh 4% PFA and 4% in sucrose in PBS for 20 min at RT. They were then permeabilized with 0.3% Triton-X 100 in PBS for 30 min at RT. The cells were blocked with 0.3% Triton-X 100/2% bovine serum albumin/5% NGS in PBS for 30 min, then incubated for 48 h at RT with mouse v-SNARE Ykt6p antibody (Santa Cruz Biotechnology, sc-365732), rabbit anti-Ykt6 primary antibody (abcam, ab236583), and purified mouse anti-GM130 primary antibody (BD Biosciences, 610822) at 1:200 dilution, or rabbit anti-protein disulfide isomerase primary antibody (Cell Signaling Technology, 3501), all at 1:100 dilution in the blocking buffer. Anti-MAP2 chicken antibody (abcam, ab318993) at 1:10,000 dilution was used to identify neurons. For synapsin I and PSD95, rabbit synapsin-1 (D12G5) primary antibody (Cell Signaling Technology, 5297) and mouse anti-PSD95 primary antibody (Invitrogen, MA1-045) were used at 1:450 and 1:400 dilution, respectively.

After primary incubation, cells were washed three times with 0.05% Tween-20 in PBS for 15 min each, then with PBS twice for 15 min each, and then incubated with then labeled with goat anti-mouse Alexa Fluor 647 secondary antibody (Invitrogen, A-21235) or goat anti-rabbit Alexa Fluor 405 secondary antibody (Invitrogen, A-21245) at 1:5000 dilution and goat anti-chicken Alexa Fluor 488 secondary antibody (Invitrogen, A-11039) at 1:400 dilution in the blocking buffer for 2 h at 4 °C. The coverslips were then washed three times with 0.05% Tween-20 in PBS for 15 min each, and then twice in PBS for 15 min each, and mounted with Prolong Gold Antifade mountant. The cells were imaged using Nikon A1R GaAsP point-scanning laser confocal microscope using 63× oil-immersion objective (NA = 1.4) with z-series of 10 to 20 images, taken at 0.3 μm intervals, with 2048 × 2048 pixel resolution. Only cells positive for MAP2 were acquired.

### Sholl analysis

Scholl Analysis was performed in cultured rat primary hippocampal cultures. Neurons were plated and allowed to mature until DIV21 and transfected with PGK-mCherry using Lipofectamine 2000 according to manufacturer’s protocol at DIV16. At DIV21 they were washed once with PBS+, fixed with fresh 4% PFA and 4% in sucrose in PBS. They were then permeabilized with 0.3% Triton-X 100 in PBS for 7 min at RT, blocked with 3% NGS in PBS, and then stained with anti-mCherry primary antibody (Abcam ab167453) at the concentration of 1:400 diluted in 3% NGS in PBS overnight in 4 °C.

The next day, the coverslips were incubated with Goat Anti-Rabbit Alexa Fluor 594 secondary antibody (abcam, ab150080) at 1:500 concentration diluted in 3% NGS in PBS, washed 3 times with PBS, and then mounted using Prolong Gold Antifade mountant. Isolated neurons were imaged using Nikon A1R GaAsP point-scanning laser confocal microscope using 63× oil-immersion objective (NA = 1.4) with z-series of 8 to 10 images, taken at 0.3 μm intervals, with 1024 × 1024 pixel resolution. The stacks were then flattened to one image in ImageJ (https://imagej.net/ij/)and Scholl Analysis performed using the SNT plugin (ImageJ) (156) after manual tracing.

### Ykt6/organelle analysis

To measure the relative intensities of Ykt6 and the respective organelles, ImageJ was used with the Color Profiler plugin. For the soma, a line was drawn in between the beginnings of the basal dendrites of the hippocampal neurons. For the dendrites, the line was drawn across the width of the secondary dendrite, immediately after the bifurcation point.

### PSD95/synapsin distance measurement

To measure the distance between PSD95 puncta and its most proximate Ykt6 puncta, coverslips were incubated per protocol above using the mouse anti-PSD95 and the rabbit anti-Ykt6 antibodies at given concentrations. The coverslips were imaged on Nikon AXR point-scanning laser confocal microscope using 100x (NA = 1.49), with 2048 × 2048 pixel resolution and Nyquist. The images were analyzed using ImageJ (85) and colocalization by cross correlation package by McCall (157), by first constructing a mask to extrapolate the secondary dendrite, and then calculating the mean of all numerical values of the shortest distance of all PSD95 puncta with Ykt6 puncta.

### Pearson correlation coefficient calculation

Pearson correlation coefficient was calculated using ImageJ and JaCoP plugin (158).

### Spine analysis

Rat primary hippocampal neurons were transfected with PGK-mScarlet (a kind gift from Dr Peter Penzes, Northwestern University) using Lipofectamine 2000 according to manufacturer’s protocol at DIV16. At DIV21, they were washed once with PBS+ and fixed with fresh 4% PFA and 4% in sucrose in PBS. The coverslips were then mounted with Prolong Gold antifade mountant and imaged using Nikon AXR NSPARC point-scanning laser confocal microscope using 100x (NA = 1.49), with 2048 × 2048 pixel resolution and Nyquist. The images were analyzed using Dendritic Spine Counter plugin in ImageJ (https://imagej.github.io/plugins/dendritic-spine-counter).

### Synaptosomal fractionation

Synaptosomal fractionation was performed following a protocol adapted from Bermejo *et al.* (159). Adult brains were extracted in accordance with Northwestern University administrative panel on laboratory animal care postdecapitation. The brain was chopped with a razor blade and washed with ECS once before the glycine treatment. The brain was then snap-frozen with liquid nitrogen until further processing.

The fractionation was performed as follows: solutions shown in Table 1 were chilled, and HALT protease/phosphatase inhibitor (Thermo Fisher Scientific, 78440) was added at 1:100 dilution to all solutions.

**Table 1:** Reagents for synaptosomal fractionation

Solutions	Hepes-buffered sucrose
1 M Hepes pH 7.4	0.32 M sucrose in 4 mM Hepes (pH 7.4)
4 mM Hepes pH 7.4	0.8 M sucrose in 4 mM Hepes (pH 7.4)
ddH_2_O	1.0 M sucrose in 4 mM Hepes (pH 7.4)
540 mM Hepes pH7.4/2 mM EDTA	1.2 M sucrose in 4 mM Hepes (pH 7.4)

All samples and solutions were kept on ice during the procedure. The tissue was placed in a dounce homogenizer along with 1 ml of 0.32 M Hepes-buffered sucrose solution and homogenized with the motor drive set at 900 rpm (setting 7) over 30 s period with 12 strokes. The homogenizer was rinsed with deionized water and wiped dry with Kimwipes in between samples. Ten microliters of homogenate was preserved and diluted to 4 times the concentration using 0.32 M Hepes-buffered sucrose solution and then stored at −80 °C for subsequent protein quantification and Western blot analysis of the total protein fraction.

The remaining homogenate was centrifuged in a fixed angle rotor at 900*g* for 10 min at 4 °C. The supernatant (S1) was transferred to a clean Eppendorf tube, while the nuclear fraction (P1) was resuspended in 500 μl of 0.32 M Hepes-buffered sucrose and stored at −80 °C and used for Western blot analysis of the nuclear fraction.

S1 was then centrifuged at 10,000*g* for 15 min at 4 °C. The supernatant (S2) was removed and stored at −80 °C. The pellet (P2) was resuspended in 1 ml of 0.32 M Hepes-buffered sucrose solution and centrifuged at 10,000*g* for 15 min at 4 °C. The supernatant (S2′) was removed and stored at −80 °C for subsequent protein quantification and Western blot analysis of the cytosolic/light membrane fraction.

The pellet was then lysed by resuspending it in 1 ml of ddH_2_O, transferred to a glass-Teflon tissue homogenizer, and rapidly homogenized by hand with three strokes. The sample was quickly adjusted to 4 mM Hepes with 4 μl of 1 M Hepes solution, then returned to an Eppendorf tube, and the samples were then rotated at 4 °C for 30 min to complete the lysing.

The sample was then centrifuged at 21,700*g* for 24 min at 4 °C. The supernatant containing the crude vesicular fraction (S3) was stored in −80 °C, and the pellet was resuspended in 0.32 M Hepes-buffered sucrose solution. The discontinuous sucrose gradient was prepared in 4 ml open top thin wall ultra-clear tube (Beckman Coulter, 344062) by layering 1 ml each of 1.2 M Hepes-buffered sucrose solution, followed by 1.0 M Hepes-buffered sucrose solution, and then 0.8 M Hepes-buffered sucrose solution using a P1000 pipette. Finally, the sample was layered on top, and the tubes were balanced using P200 pipette and adding 0.32 M Hepes-buffered sucrose solution onto the top layer. The samples were then ultracentrifuged for 2 h at 4 °C in swinging buckets (Beckman Coulter, SW-60Ti) using Optima XE-90 ultracentrifuge (Beckman Coulter). Using an 18G needle and a 1 ml syringe, the tubes were then punctured at the 1.0 M/1.2 M Hepes-buffered sucrose solution interphase and the band (synaptic plasma membrane layer) was withdrawn. The volume was noted, and the collected layer was placed in 3.5 ml thick wall ultracentrifuge tubes (Beckman Coulter, 349622) and 2.5 times the volume of synaptic plasma membrane layer of 4 mM Hepes was added to adjust the sucrose concentration from 1.2 M to 0.32 M. The tubes were then balanced with 0.32 M Hepes-buffered sucrose solution, and the samples were ultracentrifuged in the swinging bucket rotor at 200,000*g* for 30 min at 4 °C. The supernatant was discarded, the pellet was resuspended in 100 μl of 50 mM Hepes/2 mM EDTA solution, and then stored in −80 °C until used for Western blotting.

### Cell surface biotinylation

Biotinylation procedure of cultured primary hippocampal neurons was adapted from Nair *et al.*, 2021. Rat primary hippocampal neurons were cultured as mentioned above in 10 cm culture dishes. At DIV21, cells were treated with glycine for chemical LTP induction as detailed above and then were chilled down on ice for 5 to 10 min, and then the media aspirated and washed twice with cold Earle’s buffer (140 mM NaCl, 5 mM KCl, 1.8 mM CaCl_2_, 0.8 mM MgCl_2_, 25 mM Hepes, 5 mM glucose). The buffer was then aspirated, and 10 ml of buffer containing 0.3 mg/ml EZ-Link Sulfo-NHS-SS-biotin (Thermo Fisher Scientific, 21331) was added to each dish. The dishes were incubated on ice for 10 min with gentle swirling every 2.5 min for even distribution of biotin. The cells were then washed twice with 5 ml of cold Earle’s buffer. The buffer was then removed, and 10 ml of 100 mM NH_4_Cl was added to scavenge the unbound Sulfo-NHS-SS-biotin. After 1 min, the buffer was aspirated and washed gently once with cold Earle’s buffer. The buffer was then removed and 500 μl of neuronal lysis buffer (T50 mM Tris-pH7.4, 150 mM NaCl, 1% Triton X-100, 0.1% SDS, 1% HALT protease/phosphatase inhibitor) was added. Cells were then scraped using a cell scraper, and lysate transferred to 1.5 ml Eppendorf tubes. The cells were passed through 27G syringe needles 5 times to complete lysis, and then the lysate was left on ice for 30 min, and then centrifuged for 30 min at 16,000*g*.

During centrifugation Dynabeads MyOne Streptavidin C1 (Thermo Fisher Scientific, 65001) was washed with the lysis buffer 3 times, using 300 to 500 μl of lysis buffer per wash. The beads were resuspended with 800 μl of lysis buffer, divided to 125 μl per tube. The supernatant from the lysate were transferred to fresh tubes, and protein concentration was analyzed, and protein concentration normalized to the lowest concentration. Cleared lysates were added to the beads, and the tubes were left on a rotating wheel at 4 °C for 1.5 h, and then washed three times with the lysis buffer. The initial supernatant was kept as nonmembrane bound fraction. The pellet was resuspended with 160 μl of the elution buffer (Thermo Fisher Scientific, 1863642) with 10 mM DTT added (Thermo Fisher Scientific, R0861). The final pulldown and lysate samples were then processed for Western blotting.

### Western blot

Protein concentration was analyzed with the Pierce bicinchoninic acid Protein Assay kit (Thermo Fisher Scientific, 23225). After the addition of the appropriate amount of the 6X Laemmli sample buffer (Bio-rad sab03-02) with 5% ß-mercaptoethanol (Sigma-Aldrich, M6250), protein samples were boiled and separated on precast 4 to 20% Criterion TGX Stain-free gels (Bio-Rad) and transferred to a nitrocellulose membrane (Amersham Protran 0.2 μm NC, #10600001). Membranes were blocked with 3% bovine serum albumin in 1X Tris-buffered saline (TBS) (50 mM Tris/Cl pH 7.4, 150 mM NaCl) for 1 h at RT. Membranes were subsequently immunoblotted overnight in primary antibody (anti-Ykt6p: 1:100, Santa Cruz Biotechnology, sc-365732; anti-GAPDH: 1:500, Proteintech, 60004-1-lg; anti-PSD95: 1:1000, Invitrogen, MA1-046; anti-GluA1: 1:1000, Invitrogen, MA5-27694; anti-GluA2, 1:1000, Cell Signaling Technology, 13607; anti-β tubulin: 1:1000, abcam, ab6046; anti-Na^+^/K^+^ ATPase α1: 1:500, Cell Signaling Technology, 3010) at 4 °C, shaking. The following day, membranes were washed three times with 1X TBS with 0.1% Tween for 5 min and incubated in secondary IRDye antibody at 1:15,000 dilution for 1 h shaking at RT. Membranes were washed three times with 1X TBS with 0.1% Tween for before imaging using Li-Cor Odyssey CLx Imaging System. Images were processed and quantified using Image Studio Software (LI-COR Biosciences, https://www.licorbio.com/image-studio). Biotinylated pulldowns were imaged using horseradish peroxidase–conjugated secondary antibodies (anti-mouse: Abcam, ab205719; anti-rabbit: Abcam, ab205718) at 1:5000 dilution, then developed using Radiance Q quantitative chemiluminescent horseradish peroxidase substrate (Azure Biosystems, AC2100) and the Chemidoc MP imaging system (Bio-Rad). Stripping buffer (Thermo Fisher Scientific, 21059) was used to strip the membrane and reprobe for Na^+^/K^+^ ATPase α1 for the input fraction of the biotinylation procedure as directed by the manufacturer’s protocol.

### Electrophysiology

mEPSCs were recorded from infected primary hippocampal cultures in whole-cell voltage clamp. Recordings were performed between DIV 18 to 21. The external solution contained the following (in mM): 150 NaCl, 2.8 KCl, 2 CaCl_2_, 1 MgCl_2_, 10 glucose, and 10 m Hepes (pH adjusted to 7.3 with NaOH), with D-AP5 (50 μM), PTX (50 μM), and TTX (1 μM) added. The internal solution used in recordings (in mM): 110 CsF, 30 CsCl, 10 Cs-Hepes, 5 EGTA, 4 m NaCl, and 0.5 CaCl_2_ (pH adjusted to 7.3 with CsOH). Cells were held at −70 mV in voltage clamp with an Axopatch 200B amplifier (Molecular Devices). Data were analyzed with Clampfit version 11.0.3 (Molecular Devices, https://support.moleculardevices.com/s/article/Axon-pCLAMP-10-Electrophysiology-Data-Acquisition-Analysis-Software-Download-Page).

### Electron microscopy

Samples were prepared from an adapted protocol by Arai and Waguri (160). Ten minutes at RT and 50 min at 4 °C. Cells were washed with 0.12 M phosphate buffer pH 7.4 and then treated with 1% osmium tetroxide and 1.5% potassium ferrocyanide (Sigma) in 0.12 M phosphate buffer pH 7.4. Cells were dehydrated by an ascending series of alcohol (50, 70, 80, 90, and 100%), followed by treatment with epoxy resin for 24 h. The grids were mounted on resin blocks and cured at 65 °C for 3 days. The blocks were trimmed to contain cells of interest before proceeding to ultra-thin sectioning. Resin blocks were sectioned on a Leica Ultracut UC6 ultramicrotome (Leica Inc). Sections (70 nm) were collected on 200 mesh copper-palladium grids. Ultra-thin sections were counterstained on a drop of UranyLess solution (Electron Microscopy Sciences) and 0.2% lead citrate. Grids were examined on FEI Tecnai Spirit G2 TEM (FEI Company) and digital images were captured on an FEI Eagle camera. Post-image acquisition adjustment was performed by Adobe Photoshop 2025.

Circular structures that were between 35 to 45 nm were considered as synaptic vesicles (76) and were manually counted. Those that were touching the membrane were counted as RRP, those close to the membrane but not touching were counted as recycling pool, and those distal to the membrane and the synaptic contact points were considered to be the reserve pool. Vesicular size was manually quantified using ImageJ.

### Statistical analysis

Graphpad Prism 10 (http://graphpad.com) was used to graph, organize, and perform all statistical analysis. Statistical analysis was determined using the following methods: in case of more than two conditions, Bartlett’s test was performed to test for homogeneity of variances. If the variances were heterogeneous, one-way ANOVA with Welch’s correction alongside with Dunnett’s T3 multiple comparisons test was performed; otherwise, standard ANOVA with multiple comparison was applied. In cases where two conditions were compared, F-test was performed to compare variances. If the variances were homogeneous, standard *t* test was applied; otherwise, *t* test with Welch’s correction was used to examine the significance of the results. For Sholl analysis, two-way ANOVA with mixed model was performed followed by a multiple comparisons test.

For the cumulative probability distribution, the event values were binned to construct the cumulative probability, and Kolmogorov-Smirnov test with Bonferroni correction was applied to examine whether the distribution differed significantly from each other.

## Data availability

All data are contained within the article.

## Supporting information

This article contains supporting information.

## Conflict of interest

The authors declare that they have no conflicts of interest with the contents of this article.
