# Supplementary material for: The SNARE protein Ykt6 drives insertion of the GluA1 and GluA2 glutamate receptors at synaptic spines during long-term potentiation
Source: J Biol Chem. 2025 Aug 19;301(10):110613. doi: 10.1016/j.jbc.2025.110613 (PMC12475530; doi:10.1016/j.jbc.2025.110613)
Supplement: Supporting Figures [file mmc1.pdf]

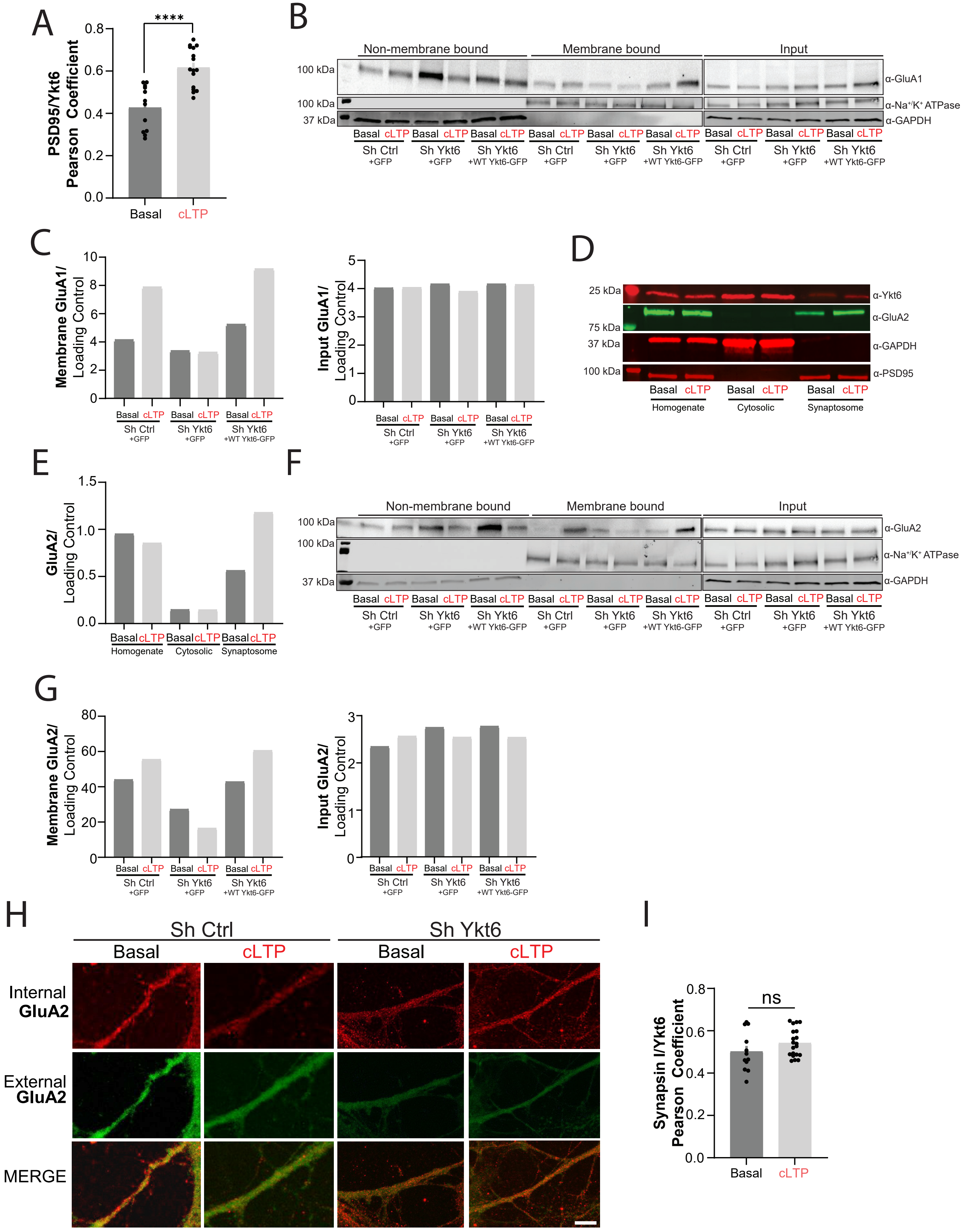

Supplemental Figure 1.

Supplemental Figure 1. A) Primary pyramidal hippocampal neurons were exposed to extracellular solution (ECS) as basal condition or glycine for chemical long-term potentiation (cLTP) at DIV21 and immunostained with the postsynaptic marker, postsynaptic density 95 (PSD95) and Ykt6, then analysed for Pearson Coefficient in the secondary dendrites. N=3 biological replicates, 3-6 cells per replicate. Unpaired T-Test, **** $p \leq 0.0001$. **B-C)** Rat primary hippocampal neurons co-transduced with 3 different conditions: 1) Sh Ctrl + GFP, 2) Sh Ykt6 + GFP and 3) Sh Ykt6 + WT Ykt6-GFP, were treated with either Earle's buffer for basal condition or glycine for cLTP, then cell-surface biotinylated and pulled down with streptavidin to isolate membrane-bound proteins. Representative western blot for membrane, non-membrane bound and input fractions for GluA1, Na^+/K^+ ATPase as a membrane marker and GAPDH as a cytosolic marker **(B)**. Quantitation of membrane-bound and input GluA1 for each condition **(C). D-E)** Adult rat brain tissue was exposed to glycine for chemical long-term potentiation (cLTP) or extracellular solution for basal condition (ECS), fractionated to cytosolic and synaptic fractions and immunoprobed for Ykt6, GluA2, GAPDH and PSD95; GAPDH serves as a loading control for the homogenate and the cytosolic fractions, and PSD95 serves as a loading control for the synaptosomal fraction. Representative western blot **(D)** and quantitation of GluA2 **(E)** levels over their respective loading controls. N=1. α-Ykt6, α-GAPDH, and α-PSD95 images reused from Figure 3A. **F-G)** Rat primary hippocampal neurons co-transduced with 3 different conditions: 1) Sh Ctrl + GFP, 2) Sh Ykt6 + GFP and 3) Sh Ykt6 + WT Ykt6-GFP, were treated with either Earle's buffer for basal condition or glycine for cLTP, then cell-surface biotinylated and pulled down with streptavidin to isolate membrane-bound proteins. Representative western blot for membrane, non-membrane bound and input fractions for GluA2, Na^+/K^+ ATPase as a membrane marker and GAPDH as a cytosolic marker **(F)**. α-Na^+/K^+ ATPase and α-GAPDH images for input lanes reused from S1B. Quantitation of membrane-bound and input GluA2 for each condition **(G)**. N=1. **H)** Representative images of secondary dendrites from the rat primary hippocampal neurons transduced with Sh Ctrl, or Sh Ykt6 under basal and cLTP conditions and then immunolabelled for external and internal GluA2. Internal GluA2 in red, external GluA2 in green. Scale bar, 10μm. **I)** Primary pyramidal hippocampal neurons were exposed to ECS for basal condition or glycine for cLTP at DIV21 and immunostained with the presynaptic marker, Synapsin I and Ykt6, then analysed for Pearson Coefficient in the secondary dendrites. N=3 biological replicates, 3-6 cells per replicate. Unpaired T-Test. Error bars represent standard errors of the mean (SEM).

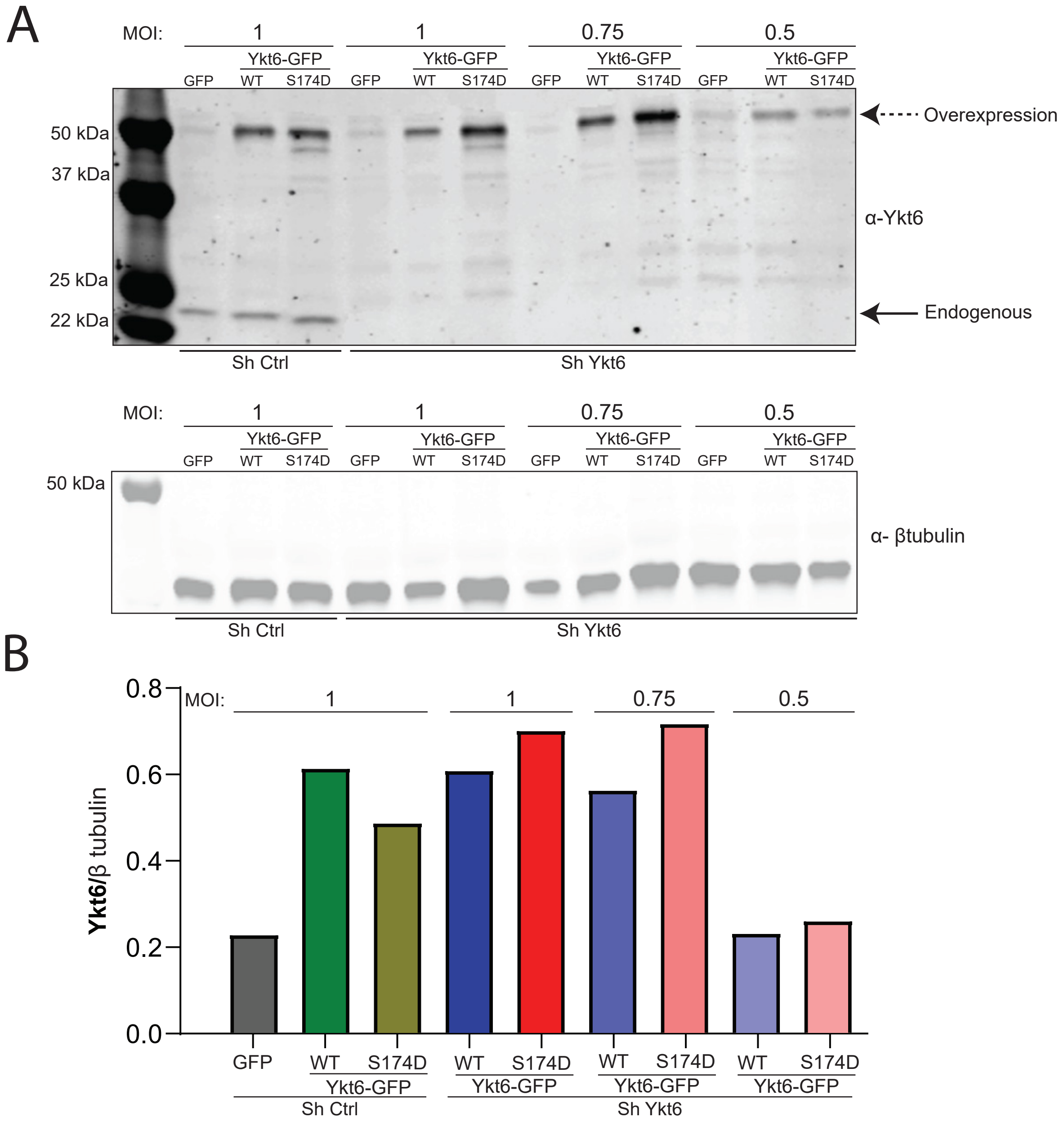

Supplemental Figure 2.

Supplemental Figure 2. Ykt6-GFP constructs successfully rescue Ykt6 knockdown. A) Representative western blot for Ykt6 expression from rat primary hippocampal neurons co-transduced with 4 different conditions: 1) Sh Ctrl + GFP, 2) Sh Ykt6 + GFP, 3) Sh Ykt6 + WT Ykt6-GFP, and 4) Sh Ykt6 + Ykt6 S174D-GFP with different MOIs for the Ykt6-GFP construct. Sh Ctrl RNA and Sh Ykt6 RNA were expressed at MOI of 1.0. Solid arrow, endogenous Ykt6; dashed arrow, exogenous Ykt6. β-tubulin serves as a loading control. **B)** Quantification of Ykt6 expression for endogenous and overexpression over loading control from (A). N=1.
